# Feasibility and impact of knowledge-based automated radiotherapy treatment planning in low- and middle-income countries

**DOI:** 10.3332/ecancer.2025.1988

**Published:** 2025-09-15

**Authors:** Awusi Kavuma, Solomon Kibudde, Yao Hao, Baozhou Sun

**Affiliations:** 1 Department of Radiotherapy, Uganda Cancer Institute, PO Box 3935, Kampala, Uganda; 2 Department of Radiation Oncology, Washington University in St. Louis, St. Louis, MO 63110, USA; 3 Department of Radiation Oncology, Baylor College of Medicine, Houston, TX, 77030

**Keywords:** radiotherapy, auto-planning, VMAT, LMIC

## Abstract

**Background and purpose:**

A high-quality treatment planning process is crucial in advanced radiotherapy techniques to ensure adequate dose to the target volume (TV) and sparing of organs at risk (OARs). This process often requires intensive labor, creating barriers for deployment in low- and middle-income countries (LMICs). We aimed to establish the feasibility of implementing knowledge-based auto-planning to facilitate clinical efficiency and increase patient throughput in an LMIC.

**Materials and methods:**

We evaluated 60 randomly selected VMAT manual plans (VMPs), including 10 for each of the following sites: head and neck, oesophagus, breast, prostate, cervical, and rectal cancers. Using the same CT-structure datasets, volumetric modulated arc therapy auto-plans (VAPs), which involved matching the patient’s CT-structure datasets with corresponding model structures before optimisation, were generated using RapidPlan^®^ knowledge-based models. The plans were compared using different dosimetric parameters, average planning times (APT) and plan scores, the latter of which was used for quantification of plan quality.

**Results:**

The APT was 29.5 ± 3.0 minutes for VAPs compared to 43.2 ± 12.0 minutes for VMPs (*p* < 0.01), an approximate 33.0% time saving. The average plan scores were 73.9% ± 9.5% and 74.8% ± 10.5% for VAPs and VMPs, respectively (*p* = 0.50). The average homogeneity and conformity indices were 0.12 ± 0.05 and 0.93 ± 0.04 for VAPs compared to 0.09 ± 0.04 and 0.94 ± 0.03 for VMPs, respectively. For prostate and breast cases, both methods achieved the rectum’s V50Gy ≤ 50% and spinal cord’s Dmax ≤ 39.0 Gy constraints; however, VAPs projected lower doses than the corresponding VMPs (16.5 ± 4.3 versus 30.6 ± 13.1: *p* = 0.01) and (6.0 ± 1.6 versus 19.6 ± 2.3: *p* < 0.01).

**Conclusion:**

The knowledge-based VAP-generated technique offers adequate dose coverage, homogeneity and conformity to the TV while sparing OARs, is less dependent on the planner’s experience, saves planning time and holds tremendous potential for improving radiotherapy workflow in LMICs.

## Introduction

Cancer continues to pose a formidable challenge to the global health landscape, and it is estimated that 18.1 million new cancer cases and almost 10.0 million cancer deaths occurred in 2020 [1]. The effects are more striking in the low- and middle-income countries (LMICs), where there are limited resources and infrastructure [2]. For most common cancers in LMICs, radiotherapy is essential for effective treatment [3]. However, the provision of high-quality radiotherapy in LMICs encounters numerous challenges, including limited access, insufficient training, high patient volumes, resource constraints and patients’ socioeconomic factors [2, 4].

A high-quality radiotherapy treatment planning (RTP) is desirable, balancing a high target dose to maximise tumour control against a sufficiently low organ-at-risk (OAR) dose to minimise toxicity [5]. The RTP process currently involves extensive manual iterations of dose-optimizing parameters by a planner, making it time-consuming and labor-intensive – achieving a high-quality plan depends on the planner’s expertise and available planning time [6].

Several centres in LMICs, Uganda Cancer Institute (UCI) inclusive, have acquired modern treatment facilities capable of performing advanced radiotherapy techniques. However, accessibility and adequate training are still limited. Major barriers hindering patient throughput include the time required to delineate targets/OAR and to generate an ideal RTP, along with limited numbers of staff [7, 8]. Volumetric modulated arc therapy (VMAT) increases the conformity of the dose to the target area, decreases the dose to OARs and effectively improves the tumour control [9]. However, VMAT planning is time-consuming and labor-intensive, its plan quality varies and it requires highly skilled staff who are not readily available in most LMICs. Although several plan optimisation procedures have been proposed, there is no clinically available robust method for developing an optimal patient-specific manually optimised VMAT treatment plan [10]. Artificial intelligence (AI) has been utilised to automate and improve RTP and to better support planners by focusing on auto-segmentation, auto-planning, and patient-specific quality assurance [6–11], and AI has made a great impact on improving planning efficiency and plan-quality consistency in high-income countries (HICs). Automated plans are faster, with consistent plan quality, minimizing human error and allowing less experienced planners to generate high-quality plans. Auto-planning has been used in HIC for several years [11, 6, 12], but it is limited in LMICs [8]. Major barriers hindering auto-plan implementation in LMICs include the need for expertise to train the models, the necessity of tuning the planning objectives in optimisation, challenges in the standardisation of the naming of structures and patient demographics that vary with different dose fractionations.

At the UCI, we began utilizing auto-segmentation in June 2022, and this development reduced the manual contouring time from ≈60 to 2 minutes per case, resulting in ≈1,500 person-hours saved annually [13]. Nevertheless, due to high patient volume and limited workforce, patient waiting times remained long after the introduction of VMAT in August 2023. We propose to use the Rapidplan^®^ model, developed in HICs, to overcome these barriers, and require only minor tuning of the objectives in optimisation to implement. This study marks the first time to implement the Rapidplan^®^ model developed for HICs to test the feasibility and impact of implementing automated VMAT planning in LMICs, by comparing it with the manually optimised planning process. The evaluation occurs before widespread usage and full deployment into clinical practice and aims to facilitate clinical efficiency and increase patient throughput. We test the hypothesis that a model trained on different demographics/dose fractions will achieve similar or better plan quality.

## Materials and methods

### Study setting

The UCI is an autonomous, specialised cancer research and treatment facility, hosting the only radiotherapy centre that receives about 2,700 new cancer patients per year. The department is equipped with three Linacs and has limited personnel numbers (three radiation oncologists, six medical physicists and nine radiation therapists). The department receives and treats the entire range of cancers, including cervical (35%), prostate (10%), breast (10%), head and neck (10%), oesophagus (5%), rectum (5%) and others (25%).

### Study design

This was a single-institution analysis, whereby we evaluated 60 randomly selected VMAT manual plans (VMPs), including 10 for each of the following sites: head and neck, oesophagus, breast, prostate, cervical and rectum cancers, all of which were previously treated between October and December 2023. These plans were replanned using VMAT auto-plans (VAPs). All the CT structure datasets were auto-segmented using an INTContour CARINA^®^

AI [14, 15] for all VMPs – to automatically generate contours for corresponding OARs and nodal regions for each of the 60 patient cases. The CTVs/planned target volumes (PTVs) were manually contoured by radiation oncologists, who also checked the OARs and nodal regions for clinical acceptability. We generated new sets of VAP with the same CT structure datasets using Online Real-time Benchmarking Informatics Technology for Radiotherapy, Rapidplan^®^ supported knowledge-based models [16]. The models for each site were imported into the Eclipse treatment-planning system (TPS), and VAPs were created and compared with VMPs by analysing different dosimetric parameters, average planning time and plan score for a quantitative measure. To evaluate the efficacy, three radiotherapy planners were timed to establish the average planning time (in minutes) it takes to complete a clinically acceptable VMP for each of the sites. The treatment planning time commenced after OARs/PTVs have been delimited and is defined as the time from starting a plan until final optimisation and dose calculation are completed. The procedure included entering the relevant parameters in the TPS, like prescription dose/fractionation, beam energy, VMAT starting and stopping gantry angles, collimator rotations, ensuring appropriate multileaf collimator coverage of the PTVs and optimisation (manually entering dose and volume constraints for different PTVs and OARs).

### Patient characteristics

Ten patients were included for each of the following sites: head and neck, oesophagus, breast, prostate, cervical and rectum cancers. For planning purposes, all patients were adequately immobilised in a supine position on a CT-simulator. The same treatment protocol/regimen was used for each of the six site plans. The delineation of OARs and dose prescriptions for the different site cases are indicated in Table 1.

### Treatment plan generation and review

Planning was done using Varian Eclipse TPS ver.16.1, for treatments with TrueBeam Linacs, calculated with AAA and used 6-/10-MV photons. The VAPs were prepared using the same energies as the VMPs. The VMPs were generated by experienced planners (more than 5 years of 3D-CRT, IMRT or VMAT planning). In most cases, two arcs were used; however, for some head and neck cases where two arcs did not yield acceptable plan quality, three arcs were used [17]. For VMPs, the PTVs, OARs and relative weights were adjusted until a clinically acceptable plan was attained and approved by the department review team that includes radiation oncologists, medical physicists and radiation therapists, with an ideal PTV D_98%_ > 95%. The VMPs involve matching the patient’s structure with corresponding model structures before optimisation. VAPs were evaluated by a medical physicist and a radiation oncologist. The VAPs were optimised once, with no further adjustments to optimisation parameters.

### Treatment plan comparison

The VMPs and VAPs were qualitatively and quantitatively evaluated. The prescriptions ensured that 100% of the prescribed doses covered 95% of the primary PTV structure and were then exported into the Eclipse plan evaluation workspace. The plans were evaluated by the comparison of different dosimetric parameters, and the evaluation of OAR doses for the various plans/sites was based on the Radiation Therapy Oncology Group guidelines [18]. The plans were also evaluated using Eclipse Scripting PlanScoreCard^®^ (Varian Medical Affairs Applied Solutions GitHub), a scoring mechanism that objectively quantifies dosimetric plan quality [19]. Examples of Dosimetric ScoreCards for different cancer sites and treatment regimens are available [20]. The plans were all standardised to a 100% score for comparison purposes, where a higher score indicates a better plan quality.

### Statistical analysis

Statistical analyses were performed to detect statistically significant differences among the 10 plans and for each of the six sites, regarding the different evaluation criteria. Statistical significance was tested using a paired two-tailed Student’s *t*‑test, assuming equal variance, and a *p*-value <  0.05 was considered statistically significant.

## Results

**Figure 1.**
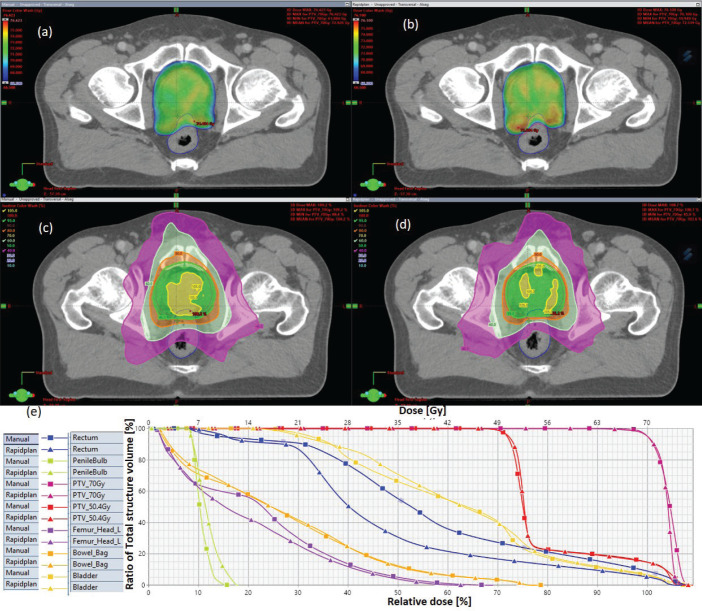
Typical two-dose level prostate SIB plan comparing: (a) 95% PTV70 for VMP, (b) PTV70 for VAP, (c) colour wash – PTV50.4 for VMP, (d) colour wash – PTV50.4 for VAP and (e) DVH for VMP versus VAP showing the two PTVs and OARs.

**Figure 2.**
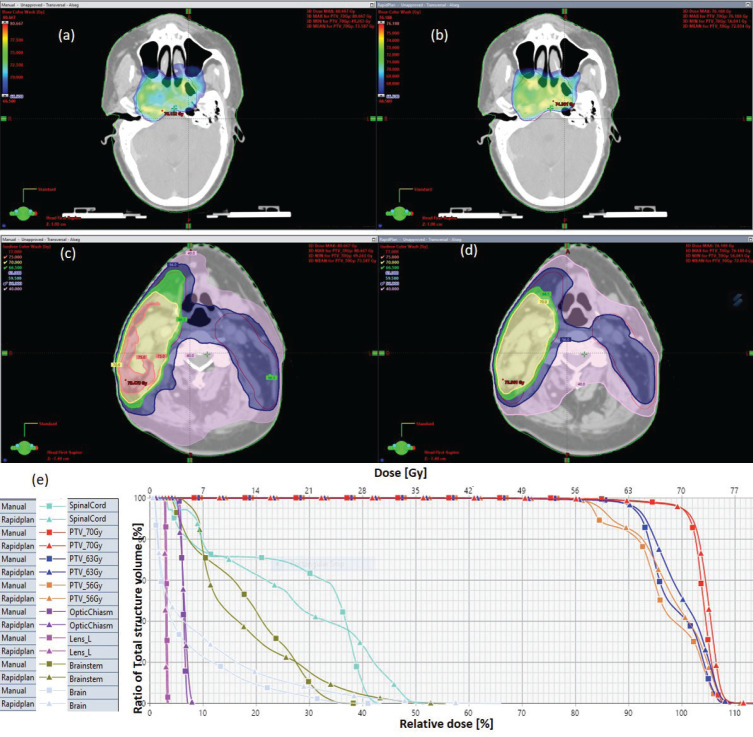
Typical three-dose level SIB plan for nasopharyngeal carcinoma, comparing: (a) 95% of PTV70 for VMP, (b) 95% of PTV70 for VAP, (c) colour wash – PTV56 for VMP, (d) colour wash – PTV56 for VAP and (e) DVH for VMP versus VAP showing the three PTVs and OARs.

The results showed that the prescription goal criterion of D98% ≥ 95% was achieved in all plans, with mean D_98%_ ranging from 95.6% to 98.1% for VAPs and from 98.2% to 99.2% (*p* = 0.060) for the VMPs. The D_2%_ ≤  107% criterion was fulfilled by 46/60 (76.7%) for VAPs (a mean value of 106.3% ± 1.5%), compared to 40/60 (66.7%) for VMPs (a mean value of 106.6% ± 1.3%) (*p* = 0.065). The dosimetric parameters analysed for the different PTV site cases are shown in Table 1. The comparison of the mean PTV (D_max_) for all cancer plans for VAPs and VMPs was not significantly different (*p* = 0.07). All plans achieved a conformity index (CI) greater than 0.90, and the mean CI for VAPs and VMPs for all sites was not statistically significant. The mean homogeneity index for VAPs and VMPs for all sites was not statistically significant, apart from the oesophagus and breast plans, where VMPs exhibited higher values. There were statistically significant differences (*p* < 0.01) in the average time to develop VAPs (29.5 ± 3.0) minutes compared to VMPs (43.2 ± 12.0) minutes, representing a 33.0% time saving. The average MU-factor was 3.0 ± 0.8 and 3.0 ± 0.2 for VAP and VMP, respectively (*p* = 0.21). For breast plans, the quality index (QI) ≤  0.3 criteria were fulfilled by both planning methods with mean values of 0.26 ± 0.06 and 0.16 ± 0.12 for VAPs and VMPs, respectively (*p* = 0.18). Figures 1 and 2 show the representative two-dose-level prostate and three-dose-level head and neck (nasopharyngeal carcinoma) simultaneous integrated boost (SIB) plans, respectively, comparing VAPs and VMPs dose distributions and corresponding dose volume histograms (DVHs). The results indicated that both computation methods achieved very good PTV coverage and OAR sparing, with VAPs showing superior protection of the rectum (Figure 1d) and larynx (Figure 2d). There were variations in the OARs’ dose constraints projected by the two methods. The dose constraints analysed for the different OARs and different site cases are shown in Table 2. There were no statistically significant differences between the VAPs and VMPs calculated results for all case sites, apart from the breast and prostate cases.

1. The breast plans: Both the VAPs and VMPs methods achieved the contralateral lung: Dmean ≤ 6.0 Gy and spinal cord: Dmax ≤ 39 Gy constraints; however, VAPs calculated lower doses than the corresponding VMP (3.6 versus 4.8: *p* = 0.01) and (6.0 versus 19.6: *p* = 0.00).
2. The prostate plans: Both the VAP and VMP methods achieved the rectum V50Gy ≤ 50% constraint; however, VAPs calculate lower doses than the corresponding VMPs (16.5 versus 30.6: *p* = 0.01).

**Figure 3.**
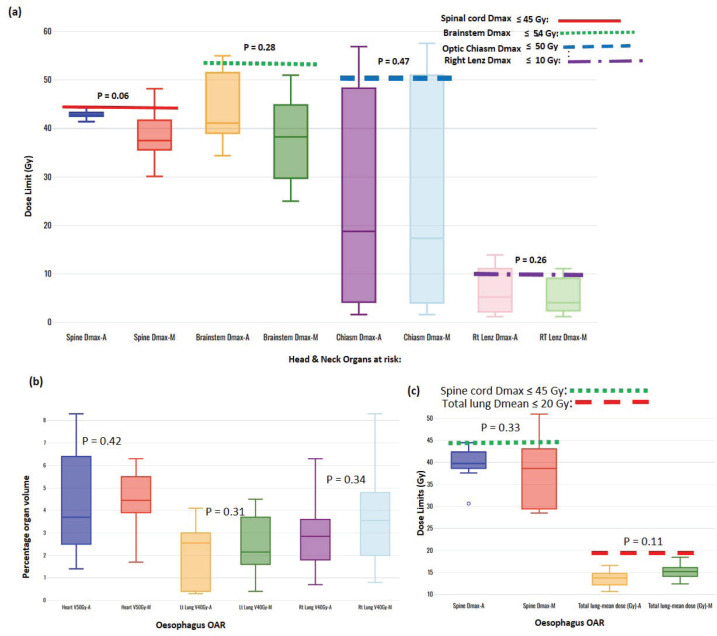
Box-and-whisker plots, comparing auto and manual planned dose limits for head and neck (a) and oesophagus (b and c) OARs.

**Figure 4.**
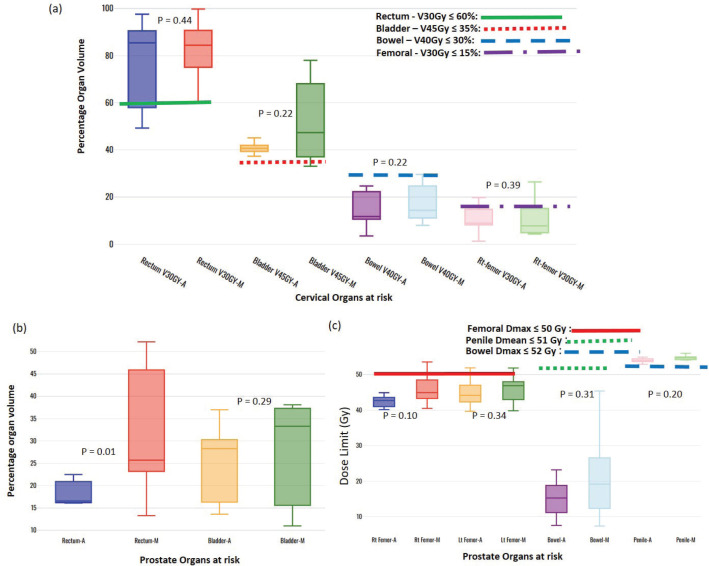
Box-and-whisker plots, comparing auto and manual planned dose limits for cervix (a) and prostate (b and c) OARs.

One of each VMP for both head and neck and oesophagus failed to fulfil the brainstem tolerance, Dmax ≤ 54 Gy, and the spinal-cord tolerance, Dmax < 50 Gy, respectively, as explicitly reflected in Figure 3a and b. For the cervical plans, both VAP and VMP methods failed to achieve the rectum: V30Gy ≤ 60% and bladder: V45Gy ≤ 35% constraints. Figure 4a and b and c explicitly reflect the trend for cervical cancer and prostate OARs, respectively.

## Discussion

Knowledge-based automated radiotherapy techniques that include auto-segmentation and auto-treatment planning have been extensively explored to address the challenges related to achieving a quality plan [21, 22]. Our results have demonstrated that the D_2%_ ≤  107% criterion, which is essential to reduce the risk of skin reactions [23, 24], was met by most of the VAPs compared to the VMPs, though the differences were not clinically significant. Keeping D_2%_ within limits ensures uniformity in dose distribution, which is essential for effective treatment. There was no significant difference between the two planning methods in terms of plan score for all sites. The mean planning time was reduced by 33.0% when using VAPs, which improves resource utilisation, especially in settings with a limited workforce. A study by Cilla *et al* [25] indicated that the mean overall planning time for auto-plans was about a third of the time needed for manual planning. The MU-factor is an important dosimetric parameter that describes the complexity of the treatment plan and delivery time. There were no statistically significant differences in the average MU-factor for both optimisation methods. Our results did not deviate from those indicated in earlier studies about the quantification of VMAT plan complexity; for TrueBeam Linacs, a threshold MU-factor of 3.62 MU/cGy is recommended, and higher values indicate a more complex plan [26].

The CI and HI are complementary tools used to compare several treatment plans for the same patient [17]. There was no significant difference in the mean HI values for all the PTV sites between the two techniques, apart from oesophagus and breast plans, where VMPs exhibited higher values. Generally, the results show that the CIs in VMPs were better than those in the VAPs’ group, in agreement with other studies [27, 28]. This is attributed to VMP’s usage of rings on the PTV to improve the conformability of the target dose and also deals with high-dose hot spots and low-dose cold spots. This option is inapplicable to VAP plans. Although there is a slight difference between these two dosimetric parameters, their dose distribution in the PTV meets the needs of clinical treatment.

Lung-D_mean_ is a parameter that monitors the risk for radiation-induced interstitial pneumonitis [29], which significantly affects the long-term survival and quality of life of patients [30]. For breast cases, mean doses in VAPs for both the ipsilateral and contralateral lung were superior to those in the VMPs, and these results are in agreement with other studies [27]. A similar observation was noted for the Lung-D_mean_ for the oesophagus cases. The heart-Dmean is a parameter that ensures continued cardiac functionality, crucial to the long-term patient's survival [31]. For the breast cases, the heart-Dmean value in VAPs was all superior compared to those in the VMPs, and the differences were statistically significant. In contrast to the oesophagus cases, the heart-Dmean values in the VMPs were all superior compared to those in the VAPs; however, the differences were statistically insignificant. The breast plan is deemed acceptable if QI ≤ 0.3 [32, 33]. VMPs' QI-values were lower than those for VAPs; however, the differences were not statistically significant (*p* = 0.18). The American Society for Radiation Oncology emphasises that when planning, the volume of breast tissue receiving >105% of the prescription dose should be as low as possible because it has been associated with adverse cosmetic effects [32, 33].

Table 2 indicates that, on average, the dose constraints for head and neck OARs like optic chiasm, eye lens, brain stem and spinal cord were adequately achieved by both methods, as explicitly reflected in Figure 3a. The same was true for prostate cases, where all pelvic OARs are within limits, apart from the bowel bag. Furthermore, although both methods achieved the rectum: V50Gy ≤ 50% constraint, VAPs predicted much lower doses than the corresponding VMPs (Dmean of 16.5 Gy versus 30.6 Gy and *p* = 0.01). This implies that the auto-plans provide better sparing of the rectum, which means that the training plans that were used to build the Rapidplan^®^ model pushed harder on the rectum than manual planners, giving a rectal protective measure. This inclination is well reflected in Figure 1 (c, d, and e) showing the dose distribution for VAPs, VMPs and DVH curves. For patients with oesophageal cancers, VAPs showed better OAR sparing than VMPs as reflected in Table 2 and Figure 3b and c, which compares VAPs and VMPs planned dose and volume limits for oesophagus OARs. The results in Table 2 further show that for both cervical and rectum plans, and for both planning methods, failed to achieve the bladder dose constraint. This is attributed to the fact that most of our patients presented with advanced cancer exhibiting extensive pelvic nodes, resulting in a large bladder volume within the PTV. Both the cervical and rectum plans had the highest average PTVs of 1,452 ± 374 and 1,602 ± 153 cm^3^, compared to 803 ± 191, 372 ± 95, 1,077 ± 280 and 1,078 ± 280, for head and neck, oesophagus, breast and prostate, respectively. A general observation from the box-and-whisker plots is that manual plans have larger standard deviations than auto plans, which reaffirms that auto-planning generates more consistent plan quality, while manual plans are dependent on the planner’s experience.

The results from this study may potentially be improved by taking into consideration aspects such as the following.

1. The standard practice for implementing Rapidplan^®^ models requires users to manually adjust optimisation parameters, with the parameters provided by the models serving as starting points, and plan quality can then be improved by further reoptimisation. The current implementation of Rapidplan^®^ applies the models directly without further adjustment that relying on the user’s experience with IMRT/VMAT. All VAPs in this study were run once. However, the current approach requires minimal experience with IMRT/VMAT planning, which is highly beneficial in LMICs. Users can achieve reasonably acceptable plans without extensive training. However, a study by Meyer *et al* [12] indicated that ≈80% of the auto-plans were acceptable without the need for secondary optimisations.
2. The time savings on VAPs can potentially increase when planners become more familiar with the optimisation process.
3. The Rapidplan^®^ models are trained by institutions in HICs. Users in LMICs do not need to retrain the model, as this process requires high-quality treatment plans, is labor-intensive and demands extensive experience. The Rapidplan^®^ models trained at different institutions may have varying dose prescriptions and treatment schemes (e.g., sequential boost versus SIB). However, our study shows that these models can be easily applied to patient populations in LMICs, despite differences in fractionations and prescriptions, and achieve plans comparable to those generated manually.

Currently, there are several published and ongoing studies on auto-planning in HICs. A study for patients who underwent breast-conserving surgery evaluated the quality of PTVs and OARs generated by manual and auto-planning modules and evaluated the feasibility of auto-planning. It was concluded that auto-planning almost achieved nearly equal quality of PTV and dose distribution as the manual module, and breast OARs were less irradiated [27].

Other authors have used the radiotherapy planning assistant, an AI software that is a machine-learning-based automated contouring and planning tool, and demonstrated that the tool can generate automated contours for CTVs, OARs and plans for a wide variety of cancer types [8, 34–36]. According to the International Organization of Medical Physics, except for North America, Europe, and a few countries in Asia, more than 75% of countries worldwide do not have accredited training programs for radiation oncology personnel [37, 38]. Hence, most regions in the world lack highly skilled professionals to produce clinical plans for radiotherapy patients. Therefore, resource-limited countries can benefit the most from using auto-planning systems.

Auto-planning is utilised in many HICs to generate high-quality treatment plans, and it outperforms manual plans in DVH metrics and blind comparisons [6, 11, 12]. However, the development of automation protocols that lead to optimal plans that adhere to institutional planning protocols remains challenging [39]. Systematic investigations of behaviour and optimal use of auto-planning are paramount to creating optimal plans. Automatic planning procedures need to be thoroughly validated, and the primary validation process is to assess how automated planning performs against manual processes by applying the automated planning routine against a representative sample of previously manually planned and treated patients [40]. This study’s primary objective was to validate automatic planning procedures and processes before full deployment into clinical practice.

In low-resource settings, where there are limited numbers of oncology personnel and expertise, auto-planning presents several advantages focused on improved efficiency and include: decreasing the time to complete a clinically acceptable radiotherapy plan, consistency (minimal variations) of treatment plans, decreasing the time it takes for patients to begin treatment (hence increased throughput as more patients are treated in a given time) and facilitating a change from inferior planning treatment/techniques like 2D/3D to more advanced IMRT/VMAT treatments [41]. The time savings may translate into cost savings. While embracing the promising future, there are challenges hindering deployment, and the implementation of automated planning in LMICs may include the following [42, 43].

(a) Validation studies are necessary to demonstrate equivalence or superiority of VAPs compared to VMPs. However, a study on fully automated CT-based cervical cancer radiotherapy by automating contouring and planning for three different treatment techniques, indicated that 87%–94% of auto-plans were found to be clinically acceptable without the need for modification [27].
(b) Conflict between tumour irradiation and normal tissue sparing – striking the right balance between automated planning and planners’ judgment is crucial; users should review and edit automatically generated contours and plans before delivery [35, 36].
(c) Education and training of radiotherapy personnel are needed to understand and effectively use automated planning systems.
(d) Developing automated protocols that result in optimal treatment plans adhering to the institute's planning guidelines.
(e) To some extent, it may impact students' learning outcomes in academic centres.
(f) Additional initial costs to obtain usage licenses.

However, spreading out the initial cost over a long time and taking into consideration the benefits outlined above, AI-based radiotherapy is cost-effective for many LMICs, as many lack adequate resources and services to manage cancer, from diagnosis, treatment planning, delivery and quality assurance [34, 44, 45]. Areas that need further investigation include: what specific improvements need to be made to the implementation of automation models to enhance plan quality? what training or resources are needed for planners in LMICs to effectively use automation techniques? and the cost-effectiveness of automated planning in terms of cost versus outcome in low-resource settings.

## Conclusion

We have compared VAPs and VMPs for different sites, with the main purpose of clearly showing that VAPs give comparable results that can be generalised to other patient populations with different cancer types, even though the models are usually trained at different institutions with different fractionation regimens. The VAP's technique offers adequate dose coverage, homogeneity and conformity to the treatment volumes for single and simultaneous-integrated-boost multiple dose levels while sparing the OARs and achieving a much-reduced planning time. The results demonstrated insignificant differences in PTV coverage and critical organs sparing. In addition, VAPs allow better dose reduction to the pelvic OARs. It is now feasible to implement auto-planning to expedite clinical efficiency and increase patient throughput, especially in LMICs, though further investigations may be required.

## Conflicts of interest

None of the co-authors declares any conflicts of interest for this research.

## Data sharing statement

The authors confirm that the data supporting the findings of this study are available within the article and/or its supplementary materials and can be shared upon request.

## Figures and Tables

**Table 1.** Dosimetric analysis of VAPs and VMPs.

Organ	OARs	Prescription Dose (Gy)	Target	Metric	Auto-plan: mean ± Std. Dev	Manual plan: mean ± Std. Dev	p-value
Head and neck	Brain, brainstem, eyes, lenses, optic nerves, cochlea, parotids, optic chiasm, spinal cord, oral cavity and mandible	Three dose levels of 56.0 Gy, 63.0 Gy and 70.0 Gy in 35 fractions using SIB technique	Plan efficacy	Plan score (%)	71.4 ± 8.1	68.4 ± 6.8	0.48
				APT (minutes)	29.5 ± 1.0	45.5 ± 13.5	0.00^*^
				MU-factor (MU/cGy)	2.9 ± 0.6	2.6 ± 0.7	0.38
			PTV70	D_max_ (%)	111.4 ± 2.6	110.8 ± 3.5	0.35
				D2%	105.3 ± 1.5	106.8 ± 2.3	0.05^*^
				D98%	94.1 ± 3.4	95.5 ± 2.6	0.16
				D50%	101.9 ± 0.4	102.3 ± 2.0	0.31
				HI	0.11 ± 0.04	0.11 ± 0.04	0.47
				CI	0.95 ± 0.04	0.91 ± 0.12	0.17
			PTV63	D_mean_ (%)	98.4 ± 1.1	97.8 ± 2.0	0.18
			PTV56	D_min_ (%)	50.6 ± 14.5	57.2 ± 14.3	0.16
Oesophagus	Oesophagus, both lungs, heart and spinal cord	50.40 Gy in 28 fractions	Plan efficacy	Plan score (%)	83.8 ± 3.1	84.1 ± 6.2	0.48
				APT (minutes)	25.5 ± 2.0	41.5 ± 12.0	0.00^*^
				MU-factor (MU/cGy)	2.8 ± 0.4	2.9 ± 0.5	0.38
			PTV50.4	D_max_ (%)	110.6 ± 2.2	109.0 ± 2.3	0.08
				D2%	104.9 ± 2.1	104.4 ± 1.8	0.27
				D98%	95.8 ± 2.4	97.6 ± 1.8	0.04^*^
				D50%	102.2 ± 2.2	101.9 ± 1.8	0.37
				HI	0.09 ± 0.02	0.07 ± 0.02	0.02^*^
				CI	0.91 ± 0.07	0.95 ± 0.06	0.13
Breast	Chest wall, axillary and supraclavicular nodal areas, contra-lateral breast, both lungs, heart and spinal cord	40.50 Gy in 15 fractions	Plan efficacy	Plan score (%)	68.2 ± 7.2	74.1 ± 7.3	0.34
				APT (minutes)	31.0 ± 1.5	44.5 ± 15.0	0.00^*^
				MU-factor (MU/cGy)	2.9 ± 0.3	3.1 ± 0.5	0.40
			PTV40.5	D_max_ (%)	115.7± 3.2	113.2± 2.2	0.03
				D2%	109.8 ± 2.7	106.6 ± 1.6	0.02
				D98%	91.0 ± 3.5	92.9 ± 1.6	0.01
				D50%	102.2 ± 4.3	101.4 ± 1.1	0.18
				HI	0.25 ± 0.11	0.14 ± 0.02	0.02
				CI	0.90 ± 0.07	0.92 ± 0.04	0.12
				QI = V105/V95	0.26 ±0.05	0.16±0.09	0.18
Prostate	Prostate, seminal vesicles, bladder, rectum, penile bulb, bowel bag, both femoral heads and nodal areas	Two dose levels: 50.4 Gy to the pelvis and 70.0 Gy in 28 fractions prostate and seminal vesicles using SIB technique	Plan efficacy	Plan score (%)	86.8 ± 5.0	83.5 ± 4.2	0.28
				APT (minutes)	31.5 ± 1.0	44.0 ± 18	0.00^*^
				MU-factor (MU/cGy)	3.0 ±0.2	2.9 ± 0.12	0.32
			PTV70	D_max_ (%)	107.9 ± 1.6	107.0 ± 0.9	0.05^*^
				D2%	103.5 ± 0.9	104.5 ± 2.1	0.10
				D98%	95.7 ± 0.9	97.7 ± 2.6	0.02^*^
				D50%	101.7 ± 0.9	102.6 ± 2.1	0.13
				HI	0.08 ± 0.01	0.07 ± 0.02	0.09
				CI	0.94 ± 0.05	0.96 ± 0.08	0.27
			PTV50.4	D_min_ (%)	44.9 ± 7.3	58.7 ± 10.9	0.01^*^
Cervical	Bladder, rectum, bowel bag, both femoral heads and nodal areas	50.0 in 25 fractions	Plan efficacy	Plan score (%)	75.1 ± 10.0	79.2 ± 12.7	0.34
				APT (minutes)	30.0 ± 1.0	42.0 ± 15.0	0.00^*^
				MU-factor (MU/cGy)	3.2 ± 0.6	3.4 ± 0.6	0.22
			PTV50	D_max_ (%)	109.2 ± 1.7	108.5 ± 1.8	0.18
				D2%	105.5 ± 1.5	105.0 ± 1.8	0.24
				D98%	96.2 ± 2.4	96.1 ± 3.1	0.47
				D50%	102.5 ± 1.9	102.1 ± 1.9	0.34
				HI	0.09 ± 0.01	0.09 ± 0.02	0.43
				CI	0.91 ± 0.09	0.91 ± 0.09	0.46
Rectum	Bladder, bowel bag, both femoral heads and nodal areas	50.0 in 25 fractions	Plan efficacy	Plan score (%)	58.1 ± 4.3	59.4 ± 4.5	0.48
				APT (minutes)	29.5 ± 1.0	43.0 ± 16.5	0.00^*^
				MU-factor (MU/cGy)	3.4 ± 1.1	3.0 ± 0.4	0.16
			PTV50	D_max_ (%)	108.9 ± 1.9	108.3 ± 4.1	0.34
				D2%	104.4 ± 0.6	104.8 ± 2.3	0.27
				D98%	96.0 ± 1.4	97.6 ± 1.4	0.01
				D50%	101.5 ± 0.1	101.8 ± 1.2	0.20
				HI	0.08 ± 0.02	0.07 ± 0.03	0.16
				CI	0.94 ± 0.05	0.96 ± 0.04	0.19
			PTV45	D_min_ (%)	69.4 ± 10.6	75.6 ± 8.2	0.07

* =*p* values < 0.05

(OARs=organs at risk, D_max_=Maximum dose, D_min_=minimum dose, D_mean_ = mean dose, p=statistical significance, MU=monitor units, PTV=Planning Target Volume, APT=Average planning time, QI=Quality Index factor=V105%/V95%, D_2%_&D_98%_ are maximum dose covering 2% and 98% of the PTV, HI= Homogeneity Index = D_2%_-D_98%_)/D_50%_, CI=Conformity Index = V98/PTV_vol_)

**Table 2.** Comparison of VAPs and VMPs OAR compliance with the dose constraints for different treatment sites.

Site	Organ Dose Constraint	Auto-plan: mean ± Std. Dev	Manual plan: mean ± Std. Dev	p-value
Head and neck	Brain stem: Dmax ≤ 54 Gy	43.6 ± 6.9	40.8 ± 13.2	0.28
	Left Lens: Dmax ≤ 10 Gy	8.7 ± 7.5	5.7± 4.0	0.14
	Right Lens: Dmax ≤ 10 Gy	6.7 ± 4.8	5.4 ± 3.7	0.26
	Spinal cord: Dmax ≤ 45 Gy	42.2 ± 2.3	39.9 ± 5.7	0.06
	Optic Chiasm: Dmax ≤ 54 Gy	24.8 ± 21.7	25.6 ± 23.4	0.47
Oesophagus	Heart: V50Gy ≤ 33%	4.3 ± 2.2	4.5 ± 1.3	0.42
	Heart: Dmean ≤ 26 Gy	20.8 ± 2.6	19.0 ± 2.9	0.08
	Combined Lungs: Dmean ≤ 20 Gy	13.5 ± 1.9	14.8 ± 2.3	0.11
	Left Lung: V40Gy ≤ 10%	2.2 ± 1.4	2.3 ± 1.4	0.31
	Right Lung: V40Gy ≤ 10%	3.1 ± 1.7	3.7 ± 2.2	0.34
	Spinal cord: Dmax ≤ 45 Gy	38.6 ± 4.9	37.3 ± 7.6	0.33
Breast	Heart: Dmean ≤ 5.0 Gy	4.0 ± 1.0	8.3 ± 1.4^**^	0.01^*^
	Combined Lungs: Dmean ≤ 10.0 Gy	10.3 ±1.3^**^	13.2 ± 0.8^**^	0.01^*^
	Ipsilateral Lung: Dmean ≤ 12.0 Gy	15.4 ± 2.0^**^	19.5 ± 1.5^**^	0.01^*^
	Contralateral Lung: Dmean ≤ 6.0 Gy	3.6 ± 1.0	4.8 ± 0.9	0.01^*^
	Contralateral breast: Dmean ≤ 3.0 Gy	2.0 ± 0.50	3.1± 1.6^**^	0.02^*^
	Spinal Cord: Dmax ≤ 39.0 Gy	6.0 ± 1.6	19.6 ± 2.3	0.01^*^
Prostate	Rectum: V50Gy ≤ 50%	16.5 ±4.3	30.6 ± 13.1	0.01^*^
	Bladder: V50Gy ≤ 55%	25.4 ± 8.9	28.0 ± 11.1	0.29
	Right Femoral head: Dmax ≤ 50 Gy	43.3 ± 3.5	45.7 ± 4.1	0.10
	Left Femoral head: Dmax ≤ 50 Gy	45.2 ± 3.7	45.9 ± 3.9	0.34
	Penile Bulb: Dmean ≤ 51 Gy	18.6 ± 12.9	24.3 ± 17.1	0.20
	Bowel Bag: Dmax ≤ 52 Gy	54.0± 0.6	54.7 ± 0.6	0.31
Cervical	Rectum: V30Gy ≤ 60%	76.7 ± 18.9^**^	78.0 ± 18.9^**^	0.44
	Bladder: V45Gy ≤ 35%	46.4 ± 12.8^**^	51.6 ± 16.5^**^	0.22
	Bowel Bag: V40Gy ≤ 30%	14.2 ± 6.9	16.7 ± 7.2	0.22
	Right Femoral head: V30Gy ≤ 15%	12.6 ± 7.6	13.8 ± 12.0	0.39
	Left Femoral head: V30Gy ≤ 15%	16.0 ± 7.2	16.3 ± 11.4	0.48
Rectum	Bladder: V40Gy ≤ 40%	68.1 ± 21.2^**^	74.9 ± 23.1^**^	0.25
	Bowel Bag: V40Gy ≤ 30%	14.2 ± 8.2	17.3 ± 10.0	0.22
	Right Femoral head: Dmax ≤ 50 Gy	42.8 ± 8.0^**^	44.2 ± 5.8	0.32
	Left Femoral head: Dmax ≤ 50 Gy	45.4 ± 6.0^**^	46.1 ± 4.0	0.39

p=statistical significance

*=p values < 0.05, ^**^ = Dose constraint not fulfilled

**Figure S1.**
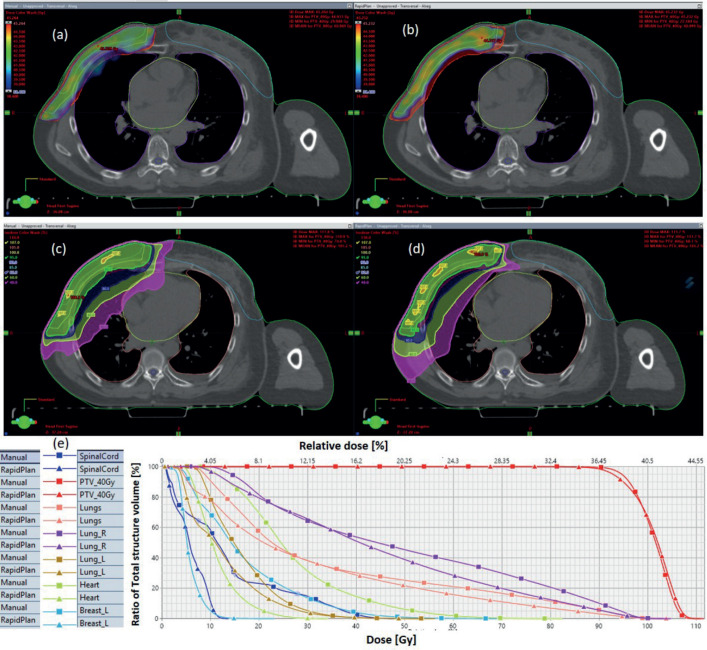
Typical right breast plan, comparing: (a) 95% of PTV40.5 for VMP, (b) 95% of PTV40.5 for VAP, (c) colour wash – PTV40.5 for VMP, (d) colour wash – PTV40.5 for VAP and (e) DVH for VMP versus VAP showing the PTVs and OARs.

**Figure S2.**
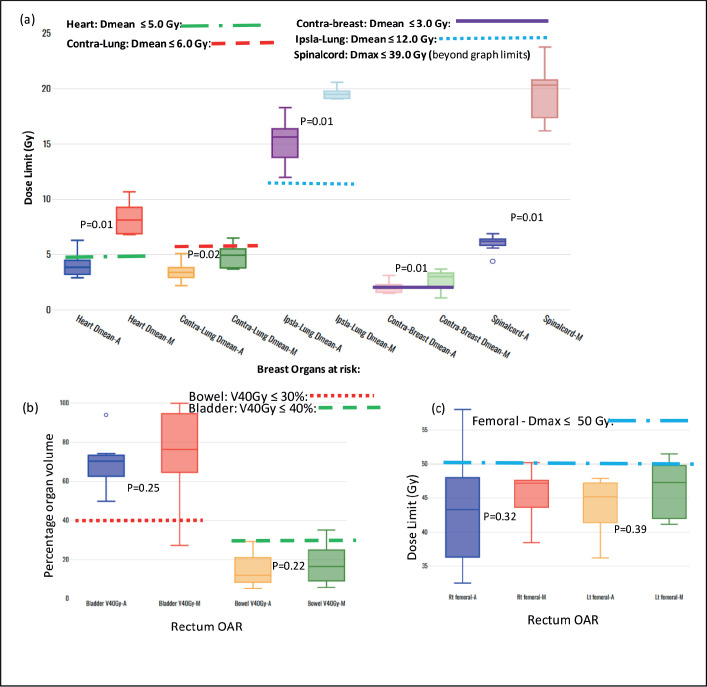
Box-and-whisker plots, comparing auto and manual planned dose limits for breast and rectum organs at risk.

## Supplementary materials

Figure S1 shows a representative breast plan, comparing VAPs and VMPs dose distributions and corresponding DVHs.

Results indicate that both computation methods achieve very good PTV coverage and OAR sparing, with VAPs showing superior protection of contralateral breast and spinal code (Figure S1).

Figure S2a,b and c compare the box-and-whisker plots for OARs for breast and rectum, cancer sites, respectively. Statistically significant differences were observed in the breast cases for heart, contra lung, and spinal code. Statistically significant differences were observed in the breast cases for heart, contra lung, and spinal code. A similar tendency is observed for the rectal plans (Figure S2b and c), where both VAP and VMP methods failed to achieve the bladder: V40Gy ≤ 40% and femoral heads: Dmax ≤ 50 Gy, failing for VAPs.

## References

[1] Sung H, Ferlay J, Siegel RL (2021). Global cancer statistics 2020: GLOBOCAN estimates of incidence and mortality worldwide for 36 cancers in 185 countries. CA Cancer J Clin.

[2] Abdel-Wahab M, Fidarova E, Polo A (2017). Global access to radiotherapy in low-and middle-income countries. Clin Oncol.

[3] Atun R, Jaffray DA, Barton MB (2015). Expanding global access to radiotherapy. Lancet Oncol.

[4] Datta NR, Samiei M, Bodis S (2014). Radiation therapy infrastructure and human resources in low-and middle-income countries: present status and projections for 2020. Int J Radiat Oncol Biol Phys.

[5] Hansen CR, Hussein M, Bernchou U (2022). Plan quality in radiotherapy treatment planning–Review of the factors and challenges. J Med Imaging Radiat Oncol.

[6] Wang C, Zhu X, Hong JC (2019). Artificial intelligence in radiotherapy treatment planning: present and future. Technol Cancer Res Treat.

[7] Grover S, Xu MJ, Yeager A (2015). A systematic review of radiotherapy capacity in low-and middle-income countries. Front Oncol.

[8] Muya S, Ndumbalo J, Kutika Nyagabona S (2023). Feasibility and clinical acceptability of automation-assisted 3D conformal radiotherapy planning for patients with cervical cancer in a resource-constrained setting. JCO Glob Oncol.

[9] Hunte SO, Clark CH, Zyuzikov N (2022). Volumetric modulated arc therapy (VMAT): a review of clinical outcomes—what is the clinical evidence for the most effective implementation?. Br J Radiol.

[10] Chen H, Craft DL, Gierga DP (2014). Multicriteria optimization informed VMAT planning. Med Dosim.

[11] Vandewinckele L, Claessens M, Dinkla A (2020). Overview of artificial intelligence-based applications in radiotherapy: recommendations for implementation and quality assurance. Radiother Oncol.

[12] Meyer P, Biston MC, Khamphan C (2021). Automation in radiotherapy treatment planning: examples of use in clinical practice and future trends for a complete automated workflow. Cancer/Radiothérapie.

[13] Kibudde S, Kavuma A, Van Rheenen J (2023). Impact of AI-based auto-segmentation on radiotherapy processes in low and middle-income countries. Int J Radiat Oncol Biol Phys.

[14] Duan J, Bernard M, Downes L (2022). Evaluating the clinical acceptability of deep learning contours of prostate and organs‐at‐risk in an automated prostate treatment planning process. Med Phys.

[15] CARINA (2020). INT Contour: Organ segmentation Powered by AI.

[16] Ray X, Truong R, Bojechko C (2023). https://orbit-rt.org/.

[17] Feuvret L, Noël G, Mazeron JJ (2006). Conformity index: a review. Int J Radiat Oncol Biol Phys.

[18] Group, R. T. O RTOG Radiation Dose Constraints.

[19] Rayn K, Clark R, Magliari A (2023). Scorecards: quantifying dosimetric plan quality in pancreatic ductal adenocarcinoma stereotactic body radiation therapy. Adv Radiat Oncol.

[20] Varian A, Siemens HC (2024). Example Dosimetric ScoreCards.

[21] Momin S, Fu Y, Lei Y (2021). Knowledge‐based radiation treatment planning: a data‐driven method survey. J Appl Clin Med Phys.

[22] Harrison K, Pullen H, Welsh C (2022). Machine learning for auto-segmentation in radiotherapy planning. Clin Oncol.

[23] Hurkmans C, Duisters C, Peters-Verhoeven M (2021). Harmonization of breast cancer radiotherapy treatment planning in the Netherlands. Tech Innov Patient Support Radiat Oncol.

[24] Wilke L, Andratschke N, Blanck O (2019). ICRU report 91 on prescribing, recording, and reporting of stereotactic treatments with small photon beams. Strahlentherapie und Onkol.

[25] Cilla S, Ianiro A, Romano C (2020). Template-based automation of treatment planning in advanced radiotherapy: a comprehensive dosimetric and clinical evaluation. Sci Rep.

[26] Nguyen M, Chan GH (2020). Quantified VMAT plan complexity in relation to measurement‐based quality assurance results. J Appl Clin Med Phys.

[27] Chen K, Wei J, Ge C (2020). Application of auto-planning in radiotherapy for breast cancer after breast-conserving surgery. Sci Rep.

[28] Krayenbuehl J, Norton I, Studer G (2015). Evaluation of an automated knowledge based treatment planning system for head and neck. Radiat Oncol.

[29] Chang DT, Olivier KR, Morris CG (2006). The impact of heterogeneity correction on dosimetric parameters that predict for radiation pneumonitis. Int J Radiat Oncol Biol Phys.

[30] Erven K, Weltens C, Nackaerts K (2012). Changes in pulmonary function up to 10 years after locoregional breast irradiation. Int J Radiat Oncol Biol Phys.

[31] Erven K, Jurcut R, Weltens C (2011). Acute radiation effects on cardiac function detected by strain rate imaging in breast cancer patients. Int J Radiat Oncol Biol Phys.

[32] Smith BD, Bellon JR, Blitzblau R (2018). Radiation therapy for the whole breast: executive summary of an American Society for Radiation Oncology (ASTRO) evidence-based guideline. Pract Radiat Oncol.

[33] Adnani N, Beyer DC, David A (2020). Minimizing the V105 in breast irradiation leads to better treatment outcomes: a retrospective study. Int J Radiat Oncol Biol Phys.

[34] Court LE, Aggarwal A, Jhingran A (2024). Artificial intelligence–based radiotherapy contouring and planning to improve global access to cancer care. JCO Glob Oncol.

[35] Rhee DJ, Jhingran A, Huang K (2022). Clinical acceptability of fully automated external beam radiotherapy for cervical cancer with three different beam delivery techniques. Med Phys.

[36] Olanrewaju A, Court LE, Zhang L (2021). Clinical acceptability of automated radiation treatment planning for head and neck cancer using the radiation planning assistant. Pract Radiat Oncol.

[37] International Organisation for Medical Physicis (IOMP) (2022). Education and Training.

[38] Elmore SNC, Polo A, Bourque JM (2021). Radiotherapy resources in Africa: an International Atomic Energy Agency update and analysis of projected needs. Lancet Oncol.

[39] Wortel G, Eekhout D, Lamers E (2021). Characterization of automatic treatment planning approaches in radiotherapy. Phys Imaging Radiat Oncol.

[40] Moore KL (2019). Automated radiotherapy treatment planning. In Seminars in Radiation Oncology.

[41] McGinnis GJ, Ning MS, Beadle BM (2022). Barriers and facilitators of implementing automated radiotherapy planning: a multisite survey of low-and middle-income country radiation oncology providers. JCO Glob Oncol.

[42] Zarepisheh M, Hong L, Zhou Y (2022). Automated and clinically optimal treatment planning for cancer radiotherapy. INFORMS J Appl Anal.

[43] Nguyen D, Lin MH, Sher D (2022). Advances in automated treatment planning. In Seminars in Radiation Oncology.

[44] Krishnamurthy R, Mummudi N, Goda JS (2022). Using artificial intelligence for optimization of the processes and resource utilization in radiotherapy. JCO Glob Oncol.

[45] Kalsi S, French H, Chhaya S (2024). The evolving role of artificial intelligence in radiotherapy treatment planning—a literature review. Clin Oncol.

